# Infrastructure and Contamination of the Physical Environment in Three Bangladeshi Hospitals: Putting Infection Control into Context

**DOI:** 10.1371/journal.pone.0089085

**Published:** 2014-02-19

**Authors:** Nadia Ali Rimi, Rebeca Sultana, Stephen P. Luby, Mohammed Saiful Islam, Main Uddin, Mohammad Jahangir Hossain, Rashid Uz Zaman, Nazmun Nahar, Emily S. Gurley

**Affiliations:** 1 Centre for Communicable Diseases, icddr, b, Dhaka, Bangladesh; 2 Global Disease Detection Program, Centers for Disease Control and Prevention (CDC), Atlanta, Georgia, United States of America; Peking Union Medical College, China

## Abstract

**Objective:**

This paper describes the physical structure and environmental contamination in selected hospital wards in three government hospitals in Bangladesh.

**Methods:**

The qualitative research team conducted 48 hours of observation in six wards from three Bangladeshi tertiary hospitals in 2007. They recorded environmental contamination with body secretions and excretions and medical waste and observed ward occupant handwashing and use of personal protective equipment. They recorded number of persons, number of open doors and windows, and use of fans. They measured the ward area and informally observed waste disposal outside the wards. They conducted nine focus group discussions with doctors, nurses and support staff.

**Results:**

A median of 3.7 persons were present per 10 m^2^ of floor space in the wards. A median of 4.9 uncovered coughs or sneezes were recorded per 10 m^2^ per hour per ward. Floors in the wards were soiled with saliva, spit, mucous, vomitus, feces and blood 125 times in 48 hours. Only two of the 12 patient handwashing stations had running water and none had soap. No disinfection was observed before or after using medical instruments. Used medical supplies were often discarded in open containers under the beds. Handwashing with soap was observed in only 32 of 3,373 handwashing opportunities noted during 48 hours. Mosquitoes and feral cats were commonly observed in the wards.

**Conclusions:**

The physical structure and environment of our study hospitals are conducive to the spread of infection to people in the wards. Low-cost interventions on hand hygiene and cleaning procedures for rooms and medical equipment should be developed and evaluated for their practicality and effectiveness.

## Introduction

Hospital-acquired infection represents a major public health concern worldwide. Hospitals have played a significant role in the spread of emerging infections. In Toronto, 77% of case patients were exposed to severe acute respiratory syndrome (SARS) in hospital settings in 2003 [1]. Hospitals in South Asia can be at particular risk for transmission of emerging infections, specifically Nipah virus [2]–[4]. For example, a large outbreak of Nipah virus was reported among healthcare workers and patients at hospitals in Siliguri, India in 2001 and evidence of Nipah virus transmission in Bangladesh has recently been reported [3]. In addition, patients in Bangladeshi hospitals face substantial risks from endemic infections; studies have shown that hospital-acquired respiratory infections occur at an incidence rate of 6.1 cases per 1000 patient-days [5], and diarrhea with an incidence rate of 3.9 cases among pediatric patients and 2.7 cases among adults per 1000 patient-days [6].

Pathogens in hospital environments can be transmitted through airborne particles, fomites, respiratory droplets or direct contact with bodily fluids [7]–[9]. International infection control guidelines exist [7], [9], but assume a level of basic infrastructure, which may not be available in many low-income settings. Understanding the context of environmental contamination in low-income hospital settings is essential to inform interventions to control the spread of hospital-acquired infection. Using data from a larger study that explored hospital-acquired respiratory illness, this paper describes the physical structure and contamination of the environment in three Bangladeshi hospitals.

## Methods

### Ethics Statement

The study protocol was approved by the Ethical Review Committee of the International Centre for Diarrhoeal Disease Research, Bangladesh (FWA # 00001468, Human Welfare Assurance # 00001822). The team obtained informed consent from hospital authorities for data collection and secured written consent from participants before conducting discussions. They observed public behavior and individuals were not identified.

### Study Site and Data Collection

The main methods of data collection for this exploratory qualitative study were structured and semi-structured direct observation. A team of three anthropologists and two sociologists, trained in qualitative research methods, collected data from March through September 2007 from one pediatric and one adult male medicine ward from each of three public tertiary teaching hospitals. They mapped the wards to describe physical layout and calculated the floor area. Next, they conducted 48 hours of observation in 22 sessions: three to four sessions in each ward. To capture variation in activities at different times of day, sessions were held during three non-overlapping periods; three hours from 9∶00 am–2∶30 pm, three hours from 3∶30 pm–9∶00 pm and one hour from 10∶00 pm–12∶30 am. Through structured direct observation, they recorded number of ward occupants-including patients, family caregivers, visitors and healthcare workers, use of fans, and number of open doors and windows at the beginning and end of each session. They also recorded frequencies of coughing and sneezing on the wards, use of personal protective equipment and handwashing. They recorded handwashing opportunities, defined as events during which ward occupant hands were contaminated with body secretions or excretions. Handwashing opportunities included points at which hands should have been washed before an activity, such as providing patient care [10] or after an activity, such as coughing or sneezing into hands [11]. Through semi-structured direct observation, the team took detailed field notes while observing the disposal of waste, the reuse of medical equipment, and the soiling with body secretions and excretions of surfaces such as floors, walls, bedding, tabletops, verandas and window grills. They also noted the presence of animals inside wards and waste disposal outside wards.

To complement observation findings, the team conducted nine focus group discussions; one with each of the groups of doctors, nurses and support staff in each hospital. They approached all the staff working in the study wards and some staff from other wards and enrolled those who consented to participate in the discussions. Each discussion included six to 11 participants and lasted for 45 to 80 minutes. The discussions were facilitated at the hospitals by NAR, RS and MSI and audio recorded.

### Data Analysis

The team expanded the observation field notes and transcribed the recorded data verbatim from discussions. NAR and RS reviewed data from observations to identify emerging themes relevant to the study objective and summarized the data according to those themes. They also reviewed focus group discussions to identify relevant data to further cross-check and complement the observations.

## Results

### Physical Environment of the Wards

#### Structure

The wards had either an open floor plan or cubicles with four-foot high walls. The floor areas ranged from 101 to 317 m^2^ (Table 1). Most of the windows in Hospital A could not be opened, while Hospitals B and C were ventilated with ceiling fans, windows that could be opened and doors on opposite sides of the rooms to allow cross-ventilation.

**Table 1:** Characteristics of six wards in three hospitals, 2007.

Characteristics	Hospital A		Hospital B		Hospital C	
	Adult	Pediatric	Adult	Pediatric	Adult	Pediatric
***Ward structure***						
Ward area for patients (m^2^)*	317	241	260	101	205	175
Number of beds	35	31	30	15	30	33
Mean distance between beds (m)	0.8	0.7	1.1	0.6	0.8	0.6
***Number of toilets***						
For doctors and nurses	1	0	1	2	2	8
For patients, visitors and support staff	4	1	4	2	4	2
***Number of functioning toilets***						
For doctors and nurses	1	0	1	2	2	8
For patients, visitors and support staff	3	1	4	2	4	2
***Number of bathrooms***						
For patients, visitors and support staff	3	1	3	2	2	2
***Number of functioning bathrooms***						
For patients, visitors and support staff	2	0	3	1	1	2
***Number of urinals***						
For patients, visitors and support staff	2	2	2	0	2	0
***Number of functioning urinals***						
For patients, visitors and support staff	2	2	2	0	2	0
***Number of handwashing stations***						
For doctors and nurses	1	0	1	2	4	6
For patients, visitors and support staff	2	2	2	2	2	0
***Number of functioning handwashing stations***						
For doctors and nurses	1	0	1	2	4	6
For patients, visitors and support staff	0	0	0	0	2	0
***Average number of persons per functioning toilet***						
Doctors and nurses	5	–	5	2	3	1
Patients, visitors and support staff	17	48	25	27	14	63
***Average number of persons per functioning handwashing station***						
Doctors and nurses	5	–	5	2	1	1
Patients, visitors and support staff	–	–	–	–	28	–

*Ward area included patient beds and nursing stations.

#### Sanitation facilities

The condition of and accessibility to sanitation facilities varied for different categories of ward occupants (Table 1). Senior doctors usually had toilets or handwashing stations with running water and soap inside their offices. Only Hospital C had toilets and handwashing stations for junior doctors. Nurses had separate toilets and handwashing stations and usually bought their own soap. In Hospital A, toilets and handwashing stations for doctors were located outside and away from the wards. In the pediatric ward of Hospital B, there was only one toilet with a handwashing station shared by nurses and junior doctors from adult and pediatric wards.

Patients, family caregivers, visitors and support staff all used patient toilets and handwashing stations. Only two of 12 patient handwashing stations had running water and the team did not observe any water stored by the handwashing stations. There was no soap at any of these stations, though some patients and caregivers brought their own soap. Patients and family caregivers used bathroom facilities for bathing, and washing clothes and utensils. They used urinals only for urinating. Non-functioning toilets, bathrooms, urinals and handwashing stations were used as waste containers.

#### Density on wards

**Figure 1:**
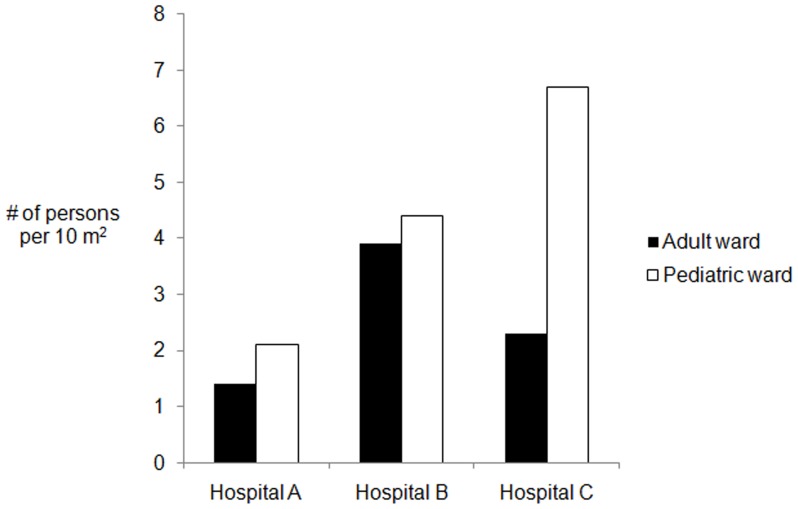
Median people present per 10 **m^2^ by type of ward in three hospitals, 2007.** **Detailed legend:** Median number of people present per 10 m^2^ by type of ward of the three hospitals, 2007.

The hospital wards were crowded with patients, caregivers, visitors and staff, especially from morning to afternoon, when doctors made their rounds and patients were admitted and discharged. An overall median of 3.7 (interquartile range [IQR]: 2.0–5.3) ward occupants were present per 10 m^2^ of floor space (Table 2). Wards in Hospitals B and C had an average of 1.2 times more patients than beds, whereas in Hospital A, the patient-to-bed ratio was approximately 0.7. When beds were unavailable, patients were cared for on mattresses or blankets on the floor near patient beds or on adjacent verandas. Pediatric wards were more crowded than adult wards (Figure 1). On a few occasions in the pediatric wards in Hospitals B and C, two or three patients shared one bed. In Hospital B, newborn babies were kept in the same ward with older children. Patients with potentially infectious diseases, such as diarrhea and encephalitis, were hospitalized in the same ward with newborn babies and patients receiving chemotherapy.

**Table 2:** Median number of people present per 10^2^ in three hospitals, 2007.

Time slot ofobservations	Median number of people (IQR*)			
	Hospital A	Hospital B	Hospital C	Overall
9∶00 am to 2∶30 pm	2.1 (1.6–2.5)	5.0 (4.4–7.9)	5.8 (4.6–8.9)	4.6 (2.6–8.2)
3∶30 pm to 9∶00 pm	1.7 (1.2–2.1)	3.6 (2.8–3.9)	4.2 (1.8–6.4)	2.4 (1.8–3.9)
10∶00 pm to 12∶30 am	1.9 (1.3–2.4)	4.1 (3.7–4.4)	3.7 (1.6–5.8)	3.1 (1.6–4.4)
Irrespective of time slot	1.9 (1.4–2.4)	4.1 (3.7–4.5)	5.2 (2.2, 6.8)	3.7 (2.0–5.3)

*IQR indicates interquartile range.

Since the hospitals were also teaching facilities, medical students accompanied senior physicians on their rounds. On one ward, 37 students accompanied two doctors for almost an hour. Students stood nearby or sat on patient beds. Below is the description of a pediatric ward from the observation notes.

The ward became crowded, even the verandas were filled with patients and caregivers. Some of the patients’ mothers had another healthy child staying in the ward. On the examination bed and nursing table, doctors and nurses quickly examined two or three patients at a time, gave medicines, injections and/or nebulizers, placed and removed canula and drew blood.

**Figure 2:**
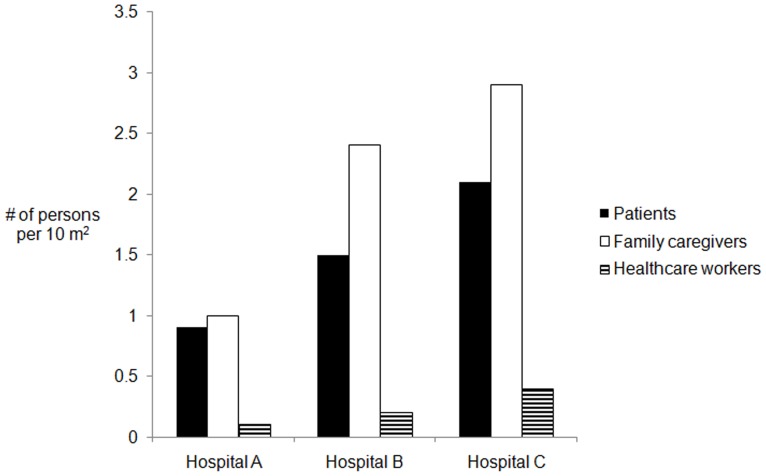
Median people present per 10 m^2^ by category of person in three hospitals, 2007. **Detailed legend:** Median number of people present per 10 m^2^ by category of person in the three hospitals, 2007.

Overall, family caregivers were most the numerous on our study wards in each hospital (Figure 2); a median of 2.1 caregivers per 10 m^2^, compared to 1.3 patients and 0.2 healthcare workers. Although these hospitals had fixed visiting hours, visitors entered wards at all times. In Hospitals A and C, vendors moved from ward to ward selling tea, water or snacks or offering haircutting services.

#### Waste disposal

Open bowls or buckets under patient beds for disposing waste (used medical supplies, patient body fluids, discarded food) were emptied into larger drums once daily by the hospital cleaners. A cleaner stated,

“We have to carry the waste [in a bucket] from the third floor on our shoulder to dump on the ground (i.e., ground floor deposited directly on the ground). There is nothing [like a trolley] to help carry the bucket… Sometimes there are holes in the bucket and waste drops down on our bodies.”

The team observed children crawling on the floor and playing with used syringes with needles. Only Hospital C had separate cardboard boxes beside the nursing stations to discard used sharps; it also had a functioning incinerator. Cleaners discarded waste on open grounds adjacent to the hospital building. City corporation vehicles removed waste from these grounds once a week. The team observed young boys and women collecting used syringes and saline bags from hospital grounds. They reported they planned to resell them.

#### Animals and insects

Feral cats were commonly observed in all wards, scavenging for food in cabinets and waste bins, climbing on patient beds and sleeping on patient bedding. The team also observed mosquitoes in the wards. All patients used mosquito nets at night in Hospitals A and C, but not in Hospital B.

### Contamination of the Environment

#### Air

Many windows and doors remained fully or partially closed. Some ceiling fans remained off every day due to electrical outages. Below is a description of ventilation in a pediatric ward.

All fans remained off, except the one in the nursing station, and most of the windows remained closed most of the time. Family caregivers explained that the children had colds, fever, breathing difficulty and pneumonia and that airflow was harmful to patients with such illnesses.

The team observed a total of 6,033 coughs and 75 sneezes in 48 hours of observation; a median of 4.9 (IQR: 4.1–7.4) uncovered coughs or sneezes per 10 m^2^ per hour per ward. Only 60 coughs and 20 sneezes were covered; four coughs were covered by a cloth and the rest by the cougher’s or sneezer’s hands. No persons were observed to wash hands after coughing or sneezing. Only one family caregiver used a cloth mask while caring for the patient.

#### Surfaces

The floors, walls, grills of windows and verandas, bedrails, nursing tabletops, bedcovers, mattresses and blankets were soiled with ward occupant body secretions and excretions. The team observed floors being soiled 125 times in 48 hours. The following excerpts illustrate the soiling of surfaces.

While setting a blood transfusion bag in a patient’s hand, the doctor accidentally dripped blood on the bed and floor. No staff cleaned it during observation hours.

While drawing gastric fluid from a patient’s stomach, fluid dripped on the bedcover and the stain remained visible two days later when the bed with the unchanged bedcover was occupied by another patient.

Cleaners swept the ward floors daily with dry brooms. Although cleaners in all hospitals reported wet mopping wards two to three times daily, the team observed daily wet mopping only in Hospital A and weekly sweeping with water in Hospital B. Cleaners also reported using disinfectant while mopping if disinfectants were available. Soiled blankets or mattresses were only shaken to remove dust after being used by one patient and then provided to another. No cleaning of window grills, bedrails, cabinets or walls was observed.

The floors of the patient toilet areas were wet, slippery and soiled with body secretions and excretions and food remnants. In one hour, the team observed seven pediatric patients urinate on the floor near the entrance of the toilet area at night since there was no light inside the toilet area.

#### Medical instruments

Doctors and nurses used the same medical instruments, such as stethoscopes, sphygmomanometers and clinical hammers, for all patients in the ward without disinfecting them between patients. Nebulizers were used 14 times and no disinfectant was observed before or after use. A doctor explained,

“The same oxygen tube is used for almost all the patients. There is only one oxygen cylinder and one mask in the ward and that mask is used for every patient.”

Thermometers, used in the mouth, were partially dipped in bottles half-filled with disinfectant after each patient use.

#### Hands

A total of 3,373 handwashing opportunities were noted during 48 hours, and occurred before or after family caregivers and healthcare workers cared for patients, after ward occupants blew their noses, coughed, sneezed, or vomited, and before ward occupants consumed food (Table 3). The most frequently observed opportunities occurred during patient feeding and physical examination (Table 3). Handwashing with soap was observed on only 32 (1% of the 273 observed handwashing events) occasions. Rinsing fingers with water before and after eating was common. Caregivers frequently touched patients and use of disposable gloves was observed only three times. The team observed only one doctor wearing gloves and a mask during rounds in Hospital C. They observed a staff member cleaning bins wearing disposable latex gloves, which he later hung on the window grill of the toilet to reuse.

**Table 3:** Handwashing opportunities observed in three hospitals, 2007.

Handwashing opportunities	Frequency
During patient care	
*Before giving food, drinks, or medicine either orally or through a nasogastric tube, before administering* *eye or ear drops, or before breastfeeding*	552
*Before and after conducting physical examination*	550
*Before and after giving injections, placing or removing canula or IV, drawing blood or other body fluids*	458
*After sponging, wiping, or massaging the body, face or head*	389
*After touching patient to provide support*	164
*After changing clothes or spreading or arranging bed sheet or oilcloth under patient*’*s body*	144
*Before and after placing or removing nasogastric tube, oxygen, nebulizer, Ambu bag*	88
*After cleaning sputum, vomit, feces, urine, anus or inserting suppository*	74
*After cleaning waste bin or emptying catheter bag*	64
*After checking temperature or pulse*	61
*Before and after cleaning patient*’*s mouth, ear, nose or eye*	56
*Before and after placing urinary catheter*	46
*Before and after dressing or touching wound*	40
*After holding or carrying biological specimens or blood bag*	22
After blowing nose, coughing, sneezing and vomiting	
*Blowing nose using hand*	79
*Coughing into hands*	56
*Sneezing into hands*	20
*Vomiting into hands*	4
Before eating	506
Total	3373

### Infection Control Policies and Logistics

No staff mentioned knowledge of any policy or written rule on infection control. All categories of staff reported inadequate supplies of cleaning and disinfection products, bed sheets, soap and hand sanitizer. Support staff reported,

“They gave us three Harpics [a brand of toilet cleaning product] last month. We must use Harpic for professors’ handwashing station daily. There are also doctors’ handwashing stations and toilets which have to be cleaned. Two of three Harpics are used for cleaning these toilets. We have to manage cleaning the five to six patient toilets with the remaining one.”

There was no autoclave in the wards and nurses reported disinfecting medical instruments by boiling, which the team also observed, or immersing in chlorine-water solution for 10 minutes. A doctor stated,

“An instrument should be boiled for at least 30 minutes. We are so loaded with patients that nurses are only dipping instruments in hot water.”

Nurses and support staff reported using antiseptic liquid or saline to wash hands on some occasions when soap was not available. Nurses reported using surgical masks while making patient beds, which the team never observed. Nurses also reported that they could change bed sheets only every one to two weeks due to inadequate supply. They mentioned family caregivers sometimes took linens soiled with patient body secretions and excretions to wash at home because water was often unavailable in the hospital. Support staff reported using gloves when caring for patients with infectious diseases like hepatitis B, but the team never observed this. Staff also reported that supplies could not be accessed in the evening or at night when the nurse-in-charge was not on ward duty.

## Discussion

Overcrowding, inadequate sanitary facilities, lack of routine cleaning, lack of basic infection control measures and improper waste management combined to create numerous opportunities for transmission of infection in the study wards. This environment posed a threat of infection to all ward occupants.

Crowding in hospitals facilitates the spread of many diseases [12], [13]. The observed crowded environment could facilitate transmission of infectious microorganisms through coughing, sneezing, talking and contact with materials and surfaces. A study conducted in a hospital in Singapore reported higher concentrations of airborne bacteria in the most densely occupied locations, such as the pharmacy and lobby, and an occupant density of 0.5 to one person per 10 m^2^ ward area [14] compared to our reported overall median of 3.7 persons per 10 m^2^. The uncontrolled flow of visitors in our study wards may influence prevalence of hospital-acquired infection, as was noted in a cross-sectional study in surgical wards in a Bangladeshi tertiary hospital [13]. Sharing beds may also facilitate disease transmission; a study reported transmission of Nipah encephalitis from a patient to a caregiver while sharing a bed [15]. A number of diseases, such as SARS, Nipah virus, tuberculosis, measles, influenza, chickenpox, meningitis, mumps and aspergillosis can be spread by airborne or droplet transmission from coughing, sneezing or inadequate ventilation [7], [8], [15], [16]. Exposure to cold air is commonly perceived to be one reason behind acute respiratory diseases in Bangladeshi children [17], hence the restricted airflow in pediatric wards, which led to inadequate ventilation. Frequent uncovered coughing and sneezing occurred among people in close physical proximity in the study wards with suboptimal ventilation, posing a risk to all ward occupants.

Hospital surfaces could be potential reservoirs of nosocomial pathogens that can survive for a few days to several months [8], [18]. Contaminated floors, taps, door handles and walls in toilets could be potential sites for colonization of pathogens and transmission through hand contact of diseases such as cholera [19], hepatitis A [20], vancomycin-resistant enterococci [21] and puerperal fever [22]. The practice of dry sweeping is strongly discouraged in global infection control recommendations, since it can aerosolize particles that may contain microorganisms [7], [9]. Medical equipment such as nebulizers [23], stethoscopes [24] and sphygmomanometers [25] that have been used on multiple patients without disinfection can also act as fomites for pathogens such as gram-negative bacilli, coagulase-negative staphylococci and methicillin-resistant *Staphylococcus aureus* (MRSA).

Most hospital infections are acquired via direct contact [8], and hand contact is a major route of acquisition [10]. Thus hand hygiene is considered to be the single most effective measure of infection control [26]. Lack of functioning and accessible handwashing stations in study wards is one reason that handwashing with soap was infrequent. In other settings, hand hygiene compliance improved after providing multiple conveniently located handwashing stations [27]–[29]. Given the continuous hand contamination during patient caregiving, waterless hand sanitizers could also play a role in hand disinfection in addition to handwashing with soap, particularly when hands are not soiled with body secretions and excretions. This alternative is more costly than soap and water and may be unacceptable to family caregivers and staff who are unfamiliar with waterless cleaning agents. However, given the logistical constraints to accessing water on these wards, the acceptability and feasibility of waterless sanitizers should be investigated as an alternative.

The presence of animals inside wards poses a threat of transmission of zoonotic diseases. In a geriatric care center, a cat was associated with an outbreak of epidemic MRSA [30]. Evidence of hospitalizations of dengue patients in Bangladesh [31], coupled with an abundance of mosquitoes in wards, creates an opportunity for nosocomial spread of dengue, a major public health concern in Bangladesh [32].

Improper handling and unsafe disposal of hospital waste is a public health concern globally [33] and in Bangladesh [34]. Particular risks include cleaning, packaging and reusing contaminated medical equipment. Disposing of used sharps in open buckets and on open grounds at these hospitals could increase the potential for transmitting HIV [35], hepatitis B [36] and hepatitis C [37] to both healthcare workers who handle waste and the general public who scavenge in open dumpsites.

This study was conducted in only three public tertiary hospitals that were not randomly chosen; therefore the findings cannot be generalized to other government tertiary hospitals, private clinics or other non-government hospitals in Bangladesh. However, our findings are consistent with other studies in Bangladeshi tertiary hospitals that have reported crowding in wards [38], improper waste disposal [34] and poor hygiene and sanitation facilities [10]. There are many hospitals in other low-income countries with similar environments [39]–[41], particularly within South Asia [42]. Since each researcher was assigned to observe and take notes on the activities of multiple persons, we assume that some events could have been missed and the frequency of events reported here could be underestimated.

## Conclusions

Public hospitals play a crucial role in ensuring healthcare services for the poor in Bangladesh [43]. Bangladesh has 17 public tertiary hospitals and a population of 150.5 million, 31% of whom are below the poverty line [44]. Our findings indicate the physical structure and environment of the three public hospitals are conducive to spreading infections to all ward occupants. With evidence of diseases like Nipah virus, avian influenza and H1N1 in this country, this environment creates a regional and global risk for wider transmission of emerging infections. Unlike hospitals in high-income settings, hospitals in low-income settings cannot follow many international infection control recommendations due to resource constraints. Furthermore, it is possible that management that extracts unofficial fees in exchange for services, commodities and access may have a vested interest to maintain such poor conditions in some of these public facilities [45]. Interventions focused solely on education or training are unlikely to improve infection control in these hospitals; interventions should also aim to improve infrastructure and to establish administrative initiatives, such as developing and implementing infection control guidelines, monitoring of routine cleaning and providing incentives for infection control activities among healthcare staff. Low-cost interventions on hand hygiene and cleaning procedures for rooms and medical equipment should be developed and evaluated for their practicality and effectiveness.
